# Improving Community Healthcare for Patients with Parkinson's Disease: The Dutch Model

**DOI:** 10.1155/2012/543426

**Published:** 2012-02-08

**Authors:** S. H. J. Keus, L. B. Oude Nijhuis, M. J. Nijkrake, B. R. Bloem, M. Munneke

**Affiliations:** ^1^Department of Neurology, Nijmegen Centre for Evidence Based Practice, Radboud University Nijmegen Medical Centre, P.O. Box 9101, 6500 HB Nijmegen, The Netherlands; ^2^Department of Neurology, Donders Institute for Brain, Cognition and Behaviour, Radboud University Nijmegen Medical Centre, The Netherlands; ^3^Department of Rehabilitation Medicine, Nijmegen Centre for Evidence Based Practice, Radboud University Nijmegen Medical Centre, The Netherlands; ^4^IQ Scientific Institute for Quality of Healthcare, Nijmegen Centre for Evidence Based Practice, Radboud University Nijmegen Medical Centre, The Netherlands

## Abstract

Because of the complex nature of Parkinson's disease, a wide variety of health professionals are involved in care. Stepwise, we have addressed the challenges in the provision of multidisciplinary care for this patient group. As a starting point, we have gained detailed insight into the current delivery of allied healthcare, as well as the barriers and facilitators for optimal care. To overcome the identified barriers, a tertiary referral centre was founded; evidence-based guidelines were developed and cost-effectively implemented within regional community networks of specifically trained allied health professionals (the ParkinsonNet concept). We increasingly use ICT to bind these professional networks together and also to empower and engage patients in making decisions about their health. This comprehensive approach is likely to be feasible for other countries as well, so we currently collaborate in a European collaboration to improve community care for persons with Parkinson's disease.

## 1. Background

**Figure 1:**
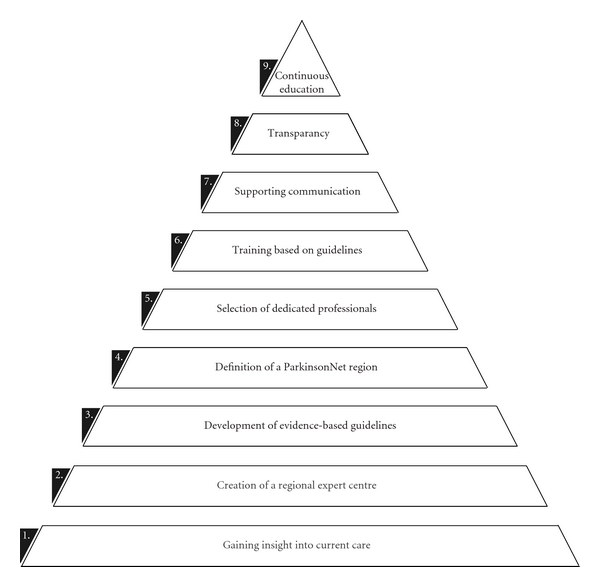
Steps of the Dutch model to improve community healthcare for Parkinson's disease.

The number of patients with Parkinson's disease (PD) and related forms of parkinsonism is increasing in all ageing societies [1]. In Western Europe's five, and the world's 10 most populous nations, the number of individuals with PD over the age of 50 was between 4.1 and 4.6 million in 2005; these numbers will be doubled by 2030 [1]. Likewise, the costs related to PD care will increase dramatically. PD is a very complex disorder, characterised by a wide array of both motor and nonmotor problems for which medical care alone is insufficient [2–6]. As a reflection of this complexity, no less than 18 different disciplines (e.g., physiotherapy and psychology) may be involved in PD care [7–9]. However, patients often have no access to the allied healthcare required [10]. Moreover, the involvement of various disciplines requires close collaboration and integration of medical and nonmedical care. Great challenges remain in the way multidisciplinary care is best realized for Parkinson patients. But, where to start? The purpose of this paper is to share the various steps we have taken (Figure 1), as they are likely to be feasible for application in other countries.

## 2. Stepwise Improvement of Community Health Care

**Step 1 (*gaining insight into current care*)**

Detailed insight into the current provision of allied healthcare, as well as barriers and facilitators for optimal care, was lacking. As a first step, we therefore aimed to evaluate current care. Surveys involving more than 500 PD patients and 300 allied health professionals were used to gain this insight [11, 12]. The results revealed that on average therapists treated as few as three individual PD patients a year. Therapists also reported that they had only limited expertise in treating PD. A major barrier for improvement was the absence of guidelines to support these therapists in providing optimal treatment. Most patients were referred by their neurologist. However, referring physicians had no information about the benefits of, for example, physiotherapy in PD, and were unable to find therapists with PD-specific interest or expertise. Finally, therapists of different disciplines (e.g., speech and language therapy, occupational therapy, and physiotherapy) were often unaware of each other's treatment possibilities. Moreover, communication about a common patient (e.g., on treatment goals and timing of interventions) was very poor. As trends have been found towards positive effects of integrated care programs in the chronically ill [13] this needed to be improved. Therefore, our next step was to create a regional Parkinson, multidisciplinary expert centre.

**Step 2 (*creation of a regional expert centre*)**

In order to offer expert care to PD patients and their carers, a regional Parkinson's expert centre was initiated. The centre serves as a tertiary referral centre for a large catchment area, by offering critical revision of the diagnosis (if needed supported by ancillary investigations), recommendations with respect to drug treatment and stereotactic neurosurgery, and individually tailored multidisciplinary treatment advice. The centre also initiates and coordinates clinical trials and disseminates the newly acquired knowledge. The centre is part of the neurology department of one of the eight Dutch university medical centres. Several comparable initiatives have meanwhile arisen across the country.

**Step 3 (*development of evidence-based guidelines*)**

Next, we started to develop evidence-based guidelines for allied healthcare. As physiotherapy is the most applied allied healthcare discipline in PD care, we first developed a guideline that targeted physiotherapy for PD. Conform international standards for guideline development, practice recommendations were developed based on the results of a systematic literature review, clinical expertise, and patient values. The recommendations were graded according to the level of evidence available [14]. The guideline was authorized and distributed by our national professional organisation for physiotherapy, the Royal Dutch Society for Physical Therapy (KNGF). Likewise, guidelines for speech and language therapy and for occupational therapy were developed, authorized, and distributed [15]. For other disciplines, for example, dieticians, the development of guidelines has recently started. The guidelines provide decision support for everyday clinical practice.

## 3. ParkinsonNet

As dissemination does not automatically lead to implementation, we developed a multifaceted implementation strategy: ParkinsonNet. ParkinsonNet not only aims to implement the guidelines, but also to reorganise allied healthcare to increase the patient volume of therapists, make expert healthcare professionals visible to other professionals as well as to patients, and support communication amongst health care professionals involved in PD care as well as between professionals and patients. To set up a ParkinsonNet, first a region needed to be defined.

**Step 4 (*definition of a parkinsonNet region*)**

Members of the ParkinsonNet team (at the regional expert centre) together with the coordinating neurologist of a general hospital defined the catchment area for which the ParkinsonNet needed to be developed. Within this geographic area, a local community of allied health professionals with Parkinson-specific expertise was created. The key points in this process were selection, training, communication, and transparency (Figure 1, steps 5 to 8).

**Step 5 (*selection of dedicated professionals*)**

To succeed in increasing the patient volume, a relatively small number of therapists were selected for each regional ParkinsonNet. To allow the networks to evolve slowly, the Dutch networks were set up with a maximum of one physiotherapist for every 20,000 residents in the specific region, and one speech and occupational therapist for every 40,000 residents. These numbers were based on the preferred patient volume as reported by therapists (i.e., 15), the estimated number of PD patients in the Netherlands, the current referral pattern of physicians (more patients are referred to physiotherapy in comparison with occupational therapy or speech and language therapy), and the number of residents in the predefined region.

For selection, all allied health professionals in a specific region were informed about the benefits, requirements, and costs for participation. In all regions, the numbers of physiotherapists interested in participation exceeded the required number, making a selection required. Professionals working in the same neighbourhood were therefore asked to arrange self-selection. Only when this was not successful, the ParkinsonNet team made the selection based on motivation, bio sketch and current function in regional PD care. In the selection process we tried to reach a good geographical dispersion in the region. This, as an evaluation under Dutch PD patients and allied health professionals, revealed that they both were prepared to travel up to 15 minutes.

**Step 6 (*training based on guidelines*)**

All allied health professionals selected for a future network participated in the same 3-day (for the first networks this was a 4-day), interactive course. Here they were trained to treat PD patients according to the evidence-based guidelines. The program entailed both mono- and multidisciplinary classes. Participating neurologists and PD nurse specialists were informed about the referral criteria and main treatment options of the allied health professions included in their regional ParkinsonNet. For physiotherapists, a guideline-based electronic patient record was developed to further support their clinical decision taking. During the course, physiotherapists were trained to use this patient record.

**Step 7 (*supporting communication*)**

Starting at the course, networking was supported. For example, professionals from a specific region shared a table during lunch and together prepared and completed educational tasks during the course. After the course, continuation of network meetings takes place during three-monthly regional seminars and a yearly ParkinsonNet congress (see Step 9). Concerning communication about common patients, the development of regional communication plan was facilitated. In addition, a secured web-based community is used to enhance communication, both within the ParkinsonNet as with hospital professionals.

**Step 8 (*transparency*)**

Patients, medical and nonmedical care professionals were informed about the ParkinsonNet. The location of the specialized therapists is visualized by web-based sources and printed folders. Moreover, structured and preferred referral to ParkinsonNet therapists by neurologists was supported by using standardized referral forms, including objective referral criteria. So, participating therapist were enabled to attract a large number of patients. A certification system, supported by professional societies, was developed to guarantee the quality of therapy provided by health professionals participating in these networks.

**Step 9 (*continuous education and exchange of knowledge*)**

Each regional ParkinsonNet organises, if needed with support by the ParkinsonNet team, three-monthly seminars to for example, practice skills, discuss cases, and to enhance (multidisciplinary) collaboration within the region. Every year, the ParkinsonNet team organises a national ParkinsonNet congress with national and international speakers and a wide variety of Parkinson's related workshops (e.g., on cognitive functioning, sleep, nutrition, and exercise and the brain). In addition, through a secured web-based community, up-to-date information is shared by the ParkinsonNet team.

## 4. Scientific Evaluation ParkinsonNet

In 2004, the first, multidisciplinary ParkinsonNet was designed and tested for its feasibility [17]. This ParkinsonNet included neurologists, a PD nurse specialist, physiotherapists, occupational therapists, and speech and language therapists. Given its feasibility and the enthusiasm of the participants, a cluster-randomized trial was designed to further evaluate ParkinsonNet. For feasibility purposes of the trial, eight ParkinsonNets were developed which only included the most used allied healthcare in PD, that is, physiotherapy. Theses clusters were compared with eight clusters where care remained unchanged. The trial, in which 699 PD patients participated, showed that the quality of care increased and the volume of patients per therapist more than doubled within as little as six months while considerably saving costs [18]. In addition, evaluation of the connectedness of healthcare professionals within the ParkinsonNet showed that especially therapists treating more than nine PD patients a year were associated with stronger connectedness with other health professionals than those treating less than 10 PD patients a year. As connectedness between professionals is known to influence clinical decision making and the coordination of patient care [19], this knowledge is of high importance to the size of future networks.

## 5. National Coverage ParkinsonNet

**Figure 2:**
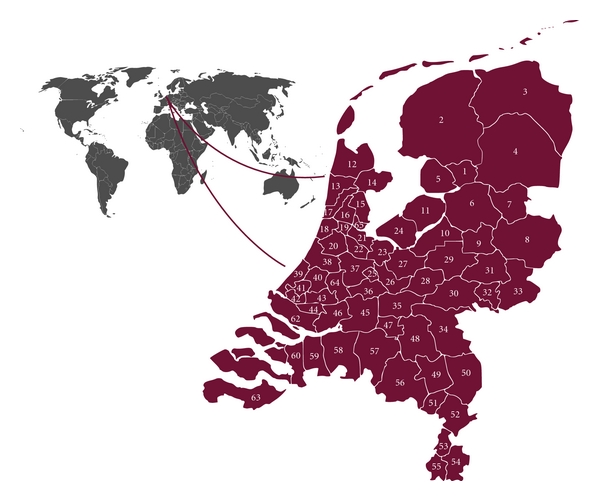
Coverage of ParkinsonNet throughout the Netherlands.

Supported by these positive results, ParkinsonNet was endorsed by professional healthcare organizations and the national patient society. In 2010 national coverage within the Netherlands was achieved by 65 unique networks (Figure 2). The size of the networks is related to the population density of the specific regions. In addition to increase in number of networks, many additional disciplines have been added to the ParkinsonNet. Currently, throughout the Netherlands, 1885 care professionals are participating, amongst which 57 neurologists, 107 PD nurse specialists, 809 physiotherapists, 317 occupational therapists, 318 speech and language therapists, 89 dieticians, 76 elderly care physicians, 62 psychologists, 31 social workers, and 4 sex therapists.

## 6. Multidisciplinary Guidelines

**Figure 3:**
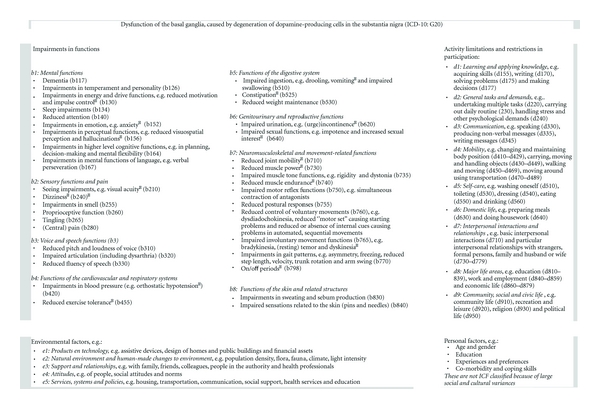
ICF model for Parkinson's disease. ICF: International Classification of Functioning, Disability and Health; numbers, for example, b770, refer to codes in the ICF Handbook [16].

In addition to the monodisciplinary guidelines, a multidisciplinary guideline has been developed in a joint collaboration among professional organizations of 18 medical professions, the patient society, and two national healthcare knowledge and quality institutes [7]. The multidisciplinary guideline includes recommendations not only for daily medical and nonmedical practice, as an update of the NICE guidelines [8], but also for network care. Specifically these recommendations, concerning collaboration, expertise, communication, and finances were lacking in the existing guidelines [20], even though they are of high importance. For example, in outpatient neurology, dissatisfaction with communication is related to noncompliance [21]. As part of the support for collaboration, the guideline provides a detailed overview of impairments, limitations, restrictions, and external factors related to PD (Figure 3) [7]. For this overview the common language of the International Classification of Functioning, Disability, and Health (ICF) was used [16]. So far, an ICF for neurology combining three neurologic disorders was available, but not for PD specific [22]. This PD-specific classification can further improve communication in relation to patient functioning between health care workers, researchers, and social policy makers.

## 7. Empowering Patients

Traditionally, the relationship between patients and their healthcare providers is fairly paternalistic, with healthcare providers making decisions (with the best intentions) and patients simply carrying out instructions. Some patients appreciate this role. However, many patients wish to have more control over their own care [23] and patient preferences may differ from what doctors focus on [24]. The guideline supports patient empowerment, for example, by teaching therapists how to get to the treatment goal in “partnership” with the patient. This, however, will not be sufficient [25]. Increasingly, the Internet can provide solutions to support patient empowerment [26]. To further support patients within ParkinsonNet regions to participate in medical decisions made about their health, we developed a web-based “portal to empower patients.” The portal provides patients with information necessary to make choices in their own health care process. For example, this includes actual and controlled information about all treatment options. In addition, patients are enabled to easily find a specialized health care professional within their community, based on transparent background information (e.g., the number of PD patients treated by a professional, or the education received to increase Parkinson-specific knowledge and expertise). Another tool for patients will be the opportunity to build their own virtual network of care providers, supporting information sharing and collaboration between all participants.

## 8. International Collaboration

As described, one of our first steps to improve PD care was the development of an evidence-based physiotherapy guideline [14]. An external quality evaluation of all Parkinson guidelines available worldwide, by the Dutch Institute for Health Care Improvement (CBO), showed that this guideline is one of the few which is of good quality [7]. In addition, to date, it is still unique in its field. As a consequence, internationally there is a lot of interest for using the guideline. The Association for Physiotherapists in Parkinson's Disease Europe (APPDE; http://www.appde.eu/) therefore endorses the guideline and its implementation.

Currently, we are updating the guideline, in a joint collaboration among 19 European physiotherapy associations, members of the European Region of the World Confederation for Physical Therapy (ER-WCPT). This will lead to a first European guideline for physiotherapy in Parkinson's disease in 2012. As a first step in the guideline development, surveys have been set out Europe-wide (*n* = 10, 000) to gain insight into current care, barriers met in delivering this care, possibilities for improvements and to identify those therapists interested to become members of a PD expert's network. Perhaps this is another first step towards improving community healthcare (Figure 1). The results of the survey will be used to develop key questions for the European guideline. In addition, the results will support the participating countries to further structure their guideline implementation plans.

At the same time, possibilities for using the ParkinsonNet concept for implementation of the guideline are being explored in several European countries as well as in the United States. The approach seems applicable to other countries with a similar population density and health care system (e.g., compensation of physical therapy). Through these future networks, also the guidelines for occupational therapy and for speech and language therapy, which are currently being translated into English in collaboration with the National Parkinson Foundation (http://www.parkinson.org/), can be implemented and thus leading to multidisciplinary Parkinson's networks in many countries.

## 9. Conclusions

Given the complex nature of PD, many disciplines will be involved in Parkinson care. Aiming for optimal Parkinson care, in the Netherlands, care for persons with Parkinson's has been changed stepwise. The Dutch approach seems applicable to other countries, be it with adaptations based on population density and health care organization.
